# Roles of MicroRNAs in Peripheral Artery In-Stent Restenosis after Endovascular Treatment

**DOI:** 10.1155/2021/9935671

**Published:** 2021-07-27

**Authors:** Mo Wang, Weichang Zhang, Lei Zhang, Lunchang Wang, Jiehua Li, Chang Shu, Xin Li

**Affiliations:** ^1^Department of Vascular Surgery, The Second Xiangya Hospital, Central South University, Changsha, Hunan, China 410011; ^2^The Institute of Vascular Diseases, Central South University, Changsha, Hunan, China 410011; ^3^State Key Laboratory of Cardiovascular Diseases, Center of Vascular Surgery, Fuwai Hospital, National Center for Cardiovascular Diseases, Chinese Academy of Medical Science and Peking Union Medical College, Beijing, China 100037

## Abstract

Endovascular repair including percutaneous transluminal angioplasty (PTA) and stent implantation has become the standard approach for the treatment of peripheral arterial disease; however, restenosis is still the main limited complication for the long-term success of the endovascular repair. Endothelial denudation and regeneration, inflammatory response, and neointimal hyperplasia are major pathological processes occurring during in-stent restenosis (ISR). MicroRNAs exhibit great potential in regulating several vascular biological events in different cell types and have been identified as novel therapeutic targets as well as biomarkers for ISR prevention. This review summarized recent experimental and clinical studies on the role of miRNAs in ISR modification, with the aim of unraveling the underlying mechanism and potential therapeutic strategy of ISR.

## 1. Introduction

Peripheral arterial disease (PAD) affects approximately 12-14% of the general population, and its prevalence increased to 15-20% in patients over 70 years. The prevalence of symptomatic intermittent claudication reaching 6% in patients over 60 years, 5-10% of patients with PAD will eventually progress to critical limb ischemia [1, 2]. PAD is a part of a diffuse atherosclerosis of the whole vasculature including coronary arteries, and it has been classified as a coronary heart disease risk equivalent, i.e., the PAD patients are at an exceptionally high risk for cardiovascular events.

There are various revascularization strategies to treat PAD, including percutaneous transluminal angioplasty (PTA), stent implantation, atherectomy, thrombectomy and interposition of the venous, and arterial or synthetic bypass graft. Endovascular treatment, including PTA and stent implantation, has become the standard approach for the femoropopliteal arterial occlusive disease [3]. Compared to PTA alone, stent implantation can prevent early elastic recoil and late constrictive remodeling. It can effectively maintain lumen volume when encountering residual stenosis or flow-limiting dissection after PTA. Therefore, stent implantation in PAD is highly prevalent especially for a longer and more complex femoropopliteal arterial lesion. However, the high incidence of in-stent restenosis (ISR) restricts the development of endovascular techniques, and it became the major cause of endovascular treatment failure and reintervention. Approximately 20-40% of PAD patients treated with bare metal stent develop ISR depending on complexity and severity, localization, and length of the lesion [4].

Bare metal stent (BMS) has been widely applied to PAD endovascular treatment; its metal scaffold implantation undoubtedly will lead to severe target vessel injury and provoke pathobiological cascade leading to neointimal hyperplasia. Compare to BMS, drug-eluted stent (DES) provides localized antiproliferative drug delivery and can effectively reduce the incidence of neointimal hyperplasia and ISR. However, the nonselective drugs also inhibit reendothelialization and healing of the injured artery and lead to fatal late phase thrombosis, therefore still far from optimized and fail to eliminate ISR [5]. As such, a cell-specific therapy targeting ISR is imperatively needed. And miRNA-based strategy may offer an alternative approach for preventing ISR. In this review, we discussed the roles of miRNAs in ISR and explored the underlying mechanism and potential therapeutic miRNA-based strategy of ISR

## 2. The Pathophysiology of In-Stent Restenosis (ISR)

**Figure 1:**
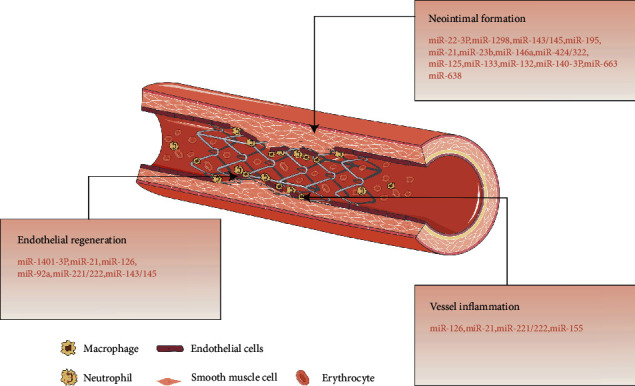
miRNA involved in in-stent restenosis (ISR). The schematic depicts the pathophysiology of ISR. Vessel inflammation, neointimal formation, and endothelial regeneration are three key processes of ISR, regulated by multiple miRNAs, respectively (see text and Table 1 for details on mechanism of all displayed miRNAs).

Once a stent was inserted into an obstructive arterial lesion, it induces an acute mechanical injury to the diseased blood vessel. The initial events immediately after stent implantation are endothelium denudation, a crush of the plaque, often accompanied with dissection into media, even adventitia, the stretch of the entire artery [6, 7]. Endothelium act as a selectively permeable barrier between the tunica media, and blood flow participates in the regulation of vascular tone and suppression of neointimal hyperplasia mainly caused by the underlying vascular smooth muscle cells (VSMCs). This alternation of endothelium is followed by the deposition of a layer of platelets and fibrin at the injured site and initiates a cascade of inflammatory response. Activated platelet expressed adhesion molecules such as P-selectin which attach to and recruit circulating leukocytes to the injured vessel. A component of leucocyte secretory granules, Mac-1 [8], promotes leukocyte that binds tightly to the surface of injured endothelial cells (ECs). The migration of leukocytes across the platelet-fibrin layer and into the tissue (i.e., leukocyte infiltration) is driven by a group of chemoattractant cytokines produced by SMCs, ECs, and resident leukocytes, such as monocyte chemoattractant protein-1 (MCP-1) and interleukin- (IL-) 8. Next is an SMC proliferation and migration phase. Growth factors, such as fibroblast growth factor (FGF), platelet-derived growth factor (PDGF), vascular endothelial growth factor (VEGF), and transforming growth factor-beta (TGF-*β*), released from platelets, leukocytes, and SMCs, stimulate proliferation and migration of SMC from the media into the neointima, leading to neointimal thickening consequently. The resultant neointima consists of SMCs, extracellular matrix (ECM) synthesis by SMCs, and macrophages recruited over several weeks [6, 7]. Compared to balloon angioplasty alone, stent implantation results in an increased and sustained inflammatory response and larger neointimal growth ultimately [7]. The late phase of ISR is vascular remodeling involving ECM protein degradation and resynthesis. There is a shift towards greater ECM synthesis rather than SMC proliferative activity [9]. ECM is composed of various collagen subtypes and proteoglycans and constitutes the major component of the mature restenotic plaque [10]. Besides, there is eventual reendothelialization (i.e., the regeneration and regrowth of the denuded endothelium) of at least part of the injured vessel surface. The reendothelialization is originated from remaining locally derived ECs and circulating endothelial progenitor cells from the blood, and it is always found to be inadequate in terms of both barrier integrity and functionality with impaired endothelium-dependent vasodilation and increased permeability [11]. An integrated view of pathophysiology of ISR has been shown in Figure 1.

## 3. General Introduction to MicroRNA

MicroRNAs (miRNAs) are short (17-25 nucleotides long), single-stranded, generally noncoding RNAs. Through the binding to miRNA-response elements (MREs) within the 3′-untranslated regions (3-UTRs) of target genes, miRNAs regulate their gene expression via posttranscriptional degradation or translational repression [12]. Most miRNA genes are located in intronic regions; they are commonly transcribed into primary-miRNA (pri-miRNA) by RNA polymerase II in the nucleus. Then, the enzyme complex of Drosha–Dgcr8 facilitates the processing of pri-miRNAs into a ~60–70 hairpin-structured precursor miRNA (pre-miRNA). Pri-miRNAs are transported to the cytoplasm from nucleus via exportin 5, the RanGTPdependent nuclear export factor. In the cytoplasm, pro-miRNAs are further processed by the RNAse III enzyme complex into mature duplex miRNA. One strand of the mature microRNA is incorporated into the miRNA-induced silencing complex (miRISC), while the other strand is usually degraded [13].

A single miRNA can regulate multiple genes, and a single gene can be regulated by multiple miRNAs. Moreover, miRNAs interact with each other to form a cotargeting network, which allows miRNAs to play an essential role in the powerful and fine regulation of almost every vascular biology. Numerous studies unravel miRNAs that act as potential biomarkers or therapeutic targets of many cardiovascular diseases [13, 14]. Of which, miRNAs have been associated with ISR-related process, such as neointimal hyperplasia, inflammatory response, and regeneration of endothelial layer (Table 1 and Figure 1).

## 4. miRNAs Involved in Neointimal Formation

Restenosis is defined as the arterial wall's healing response to mechanical injury. The key pathophysiologic phenomenon responsible for restenosis after stent implantation is the neointimal formation which consists of VSMC proliferation, migration, and ECM deposition [6, 15]. The metallic scaffold of BMS prevents vessel shrinkage (i.e., elastic recoil and negative remodeling), however, induces an enhanced neointimal hyperplasia response [6].

VSMCs within adult blood vessels are highly specialized, and they play an important role in regulating blood vessel tone, blood pressure, and blood flow distribution. Under normal conditions, VSMCs proliferate at a very low rate, exhibit low synthetic activity, and express a repertoire of contractile proteins, ion channels, and signaling molecules required for its contractile function. Although fully differentiated, VSMCs retain remarkable plasticity. In response to vascular injury induced by stent implantation, VSMCs dramatically increase their rate of cell proliferation, migration, and synthetic capacity, transiently modify their phenotype to a high “synthetic” phenotype, hence starting to proliferate and migrate to the vessel interior, causing the lumen reduction. Kearney et al. [16] retrieved in-stent restenotic tissue by directional atherectomy from 10 patients after percutaneous revascularization of PAD and demonstrated that the in-stent restenotic tissue composed predominantly of SMCs. Upon resolution of injury, VSMCs reacquire their contractile phenotype [17, 18].

Among several miRNAs, the miR-143/-145 cluster is highly expressed in VSMCs and is known as an essential mediator of SMC proliferation, differentiation, and phenotypic switching [19]. miR-143 and miR-145 are located on human chromosome 5, and they were cotranscribed as a bicistronic unit. They cooperatively target a network of transcription factors, including Kruppel-like factor- (Klf-) 4, Klf-5, Elk-1, myocardin, angiotensin converting enzyme (ACE), calmodulin kinase II *δ*, fascin, and myocardin-related transcription factor-beta (MRTF-*β*) [19, 20]. Vascular injury leads to downregulation of miR-145 in VSMCs; the downregulated miR-145 results in increased Klf-5 expression, which miR-145 directly targeting, as well as decreased myocardin and VSMC differentiation markers, e.g., smooth muscle alpha-actin, calponin, and smooth muscle myosin heavy chain (SM-MHC), inhibits migration but promotes differentiation of VSMCs [21]. miR-143 also contributes to the maintenance of VSMC contractile phenotype and suppression of VSMC proliferation, mainly through targeting Elk-1 [19].

miR-195 is also involved in the modulation of VSMC physiology. The overexpression of miR-195 inhibited VSMC proliferation and migration and therefore suppressed the neointimal hyperplasia after vascular injury through downregulating Cdc42 and its downstream mediator cyclinD1 (CCND1), which are responsible for cell cycle regulation and cell growth [22]. Similarly, miR-125a-5p is downregulated after a vascular injury caused by angioplasty. Its expression level is inversely correlated to the proliferative status: when miR-125a-5p is overexpressed, proliferation and migration of VSMCs are reduced. On the other hand, miR-125a-5p is positively correlated to the expression of Alpha Smooth Muscle Actin 2 (ACTA2), Myosin Heavy Chain 11 (MYH11), and Smooth Muscle 22 alpha (SM22*α*) which characterizes the contractile phenotype of VSMCs: these marker gene expressions are decreased during VSMC phenotypic switch from contractile to synthesis. Of note, miR-125a-5p is directly target to E26 transformation specific-1 (ETS-1), an important transcriptional factor of VSMC proliferation and migration, and is crucial in PDGF-BB pathway in VSMCs. With the stimuli of vascular injury, miR-125a-5p is downregulated, and ETS-1 upregulated consequently, promoting VSMC phenotypic switch mediated by PDGF-BB, a potent mitogen for VSMCs [23, 24]. Recently, miR-125-3p downregulation was detected in VSMC of patient suffer PAD. miR-125-3p exhibits an antiproliferative effect on VSMC via targeting mitogen-activated protein kinase (MAPK) 1, a common hub of the MAPK signaling pathway and the insulin signaling pathway [25].

miR-23b is a member of gene cluster consisting of miR-23b, miR-27b, and miR-24-1 [26]. miR-23b is downregulated after vascular injury in vivo. The experimental study showed that miR-23b overexpression is responsible for the upregulation of ACTA2 and MYH11, the markers of the contractile phenotype of VSMC, and consequently inhibits VSMC proliferation, migration, and neointimal formation after stent implantation. And this effect is mediated by miR-23b directly targets to urokinase plasminogen activator (uPA), Smad3, and Forkhead box O4 (FOXO4) which are the essential modulators of VSMC proliferation, migration, and phenotypic switch [27–29]. Similarly, the other members of the cluster, miR-27b and miR-24-1, are downregulated after vascular injury, suggesting that this cluster participates in regulation of VSMC phenotypic switch [27]. miR-663 is a novel regulator of VSMCs in the phenotypic switch. Overexpression of miR-663 is associated with increased expression of VSMC contractile phenotypic markers, such as SM22*α* and MYH11. Furthermore, miR-663 effectively inhibits VSMC proliferation and migration in vitro, and vascular injury induced neointimal hyperplasia in a mice carotid artery ligation model, through targeting the transcription factor JunB and its downstream Myl9 [30].

Recent studies reveal miR-140-3p's potential as the therapeutic target for preventing ISR in PAD [31]. miR-140-3p is mainly distributed in artery media rather than in endothelium or adventitia, and it is mainly expressed in SMCs. miR-140-3p was prominently downregulated in VSMCs with ISR. Increasing expression of miR-140-3p repressed cell proliferation via directly targeting c-Myb while promoted cell apoptosis via targeting c-Myb and Bcl-2. Intriguingly, miR-140-3p does not affect cell SMC migration [31]. In vivo study showed delivery of miR-140-3p into rat carotid artery after balloon angioplasty leads to reduction of neointimal hyperplasia. Another two miRNAs downregulated in VSMC of the patient with arteriosclerosis obliterans (ASO) are miR-22-3p and miR-1298. miR-22-3p upregulation results in attenuation of VSMC proliferation and migration in vitro via directly targeting and negatively regulating high mobility group box-1 (HMGB1). Consistent with the antiproliferative and antimigrative effect of miR-22-3p in vitro, miR-22-3p inhibits neointimal hyperplasia in balloon-injured rat carotid arteries in vivo, also by targeting HMGB1 [32]. The downregulation of miR-1298 in ASO is associated with the higher DNA methylation of its upstream CpG sites. In vitro study showed that miR-1298 suppresses VSMC proliferation and migration without affecting apoptosis [33]. These cellular effects are mediated via directly targeting connexin 43 which has been proved that its downregulation suppress VSMC phenotypic switching from contractile to synthesis [34]. In vivo study confirmed that overexpression of miR-1298 inhibits neointimal hyperplasia in an injured artery.

Besides miRNAs mentioned above, which are downregulated in response to vascular injury, there are several miRNAs upregulated after stent implantation induced vascular injury. miR-21 is one of the most upregulated miRNAs. Inhibition of miR-21 reduces VSMC proliferation while enhances VSMC apoptosis via targeting phosphatase and tensin homolog (PTEN) and Bcl-2, two crucial signaling molecules involved in VSMC growth and apoptosis [35]. Utilizing a humanized in vivo model which mimics human ISR, Wang et al. demonstrate that the miR-21 expression increased during ISR. Anit-miR-21 inhibits VSMC proliferation, while miR-21 overexpression induced proproliferative response of VSMC. This effect of anti-miR-21 is modulated through derepression of PTEN. Besides, miR-21also regulates VSMC cell shape via directly targeting Tropomyosin-1 [36]. miR-146a exhibits a potent capacity to promote VSMC proliferation in vitro and neointimal hyperplasia in vivo. miR-146a targets and inhibits Klf-4, which plays an antiproliferative role in the regulation of VSMC biology, while Klf-4 binding to miR-146a promoter inhibits miR-146a transcription. This feedback loop of miR-146a and Klf-4 regulates each other's expression and VSMC proliferation. Additionally, Klf-5 competitively binds to miR-146a promoter to promote miR-146a transcription as a Klf-4 competitor [37]. Human miR-424 or its rat ortholog miR-322 (miR-424/322) upregulated in proliferative VSMC in vitro and after vascular injury in vivo; however, it exhibits antiproliferative effect. Overexpression of miR-424/332 induced an increase in the expression of several differentiation markers such as ACTA2, calponin-1, and MYH11, inhibited VSMC proliferation and migration without affecting VSMC apoptosis by directly targeting CCND1 known as a regulator of cell cycle transition, Ca2 + -regulating proteins calumenin and by indirectly targeting stromal interaction molecule 1 (STIM1). This suggested that miR-424/322 has protective effect against neointimal hyperplasia [38].

miR-133 and miR-132 expression transiently decreased in early phase and prominently increased in a late phase of vascular injury. MAPK/ERK1/2 is the major upstream signaling to regulation miR-133. ERK1/2 activation is responsible for miR-133 downregulation. Overexpression of miR-133 significantly reduces VSMC proliferation and migration, prevents VSMC phenotype switch from contractile to synthesis, and ultimately results in inhibition of neointimal hyperplasia. These effects are modulated via directly targeting to Sp-1 and moesin and repressing the expression of these two transcriptional factors since Sp-1 regulates VSMC phenotypic switch and moesin modulates VSMC migration both in vitro and in vivo [39]. Delivery miR-132 to a rat carotid artery injury model induces increasing expression of p27 and Bax which have the proapoptosis effect, as well as expression of VSMC differentiation marker smooth muscle a-actin, and decreasing expression of Bcl-2, an antiapoptotic protein. These effects are modulated by miR-132 targets to leucine-rich repeat (in Flightless 1) interacting protein-1 (LRRFIP1), which is documented to affect cancer cell proliferation and migration. miR-132 inhibits VSMC survival and growth while promotes its apoptosis and attenuates neointimal hyperplasia conclusively [40].

miR-638 is enriched in human aortic SMC. PDGF-BB stimulation, as one of the most potent stimulants for VSMC proliferation and migration, leads to sharply decreased expression of miR-638. MAPK/ERK1/2 serves as an upstream signaling molecule that involved in the regulation of PDGF-BB-induced miR-638 decreasing. Overexpression of miR-638 leads to inhibition of VSMC proliferation and migration, as well as decrease of the CCND1 expression by directly targeting NOR1 [41].

## 5. miRNAs Involved in Vessel Inflammation

Inflammation plays a pivotal role in ISR, linking early vascular injury to the eventual consequence of neointimal hyperplasia and lumen compromise. Leukocyte recruitment and infiltration are characteristics of restenosis induced by stent implantation, trigger the subsequent neointimal formation [7]. There is mounting evidence for the role of inflammation in restenosis. Moreno and colleagues found primary lesions that develop restenosis after coronary atherectomy have more macrophages and SMCs than primary lesions that do not develop restenosis, provide evidence linking leukocytes and restenosis [42]. Cipollone et al. demonstrated significantly elevated levels of MCP-1, a potent chemoattractant of monocytes, in restenotic patients after percutaneous transluminal coronary angioplasty [43]. Tenaka et al. proved a sustained upregulation of vascular adhesion molecules-1 (VCAM-1), intercellular adhesion molecule-1 (ICAM-1), and major histocompatibility complex class II antigens in a balloon injured rabbit model [44].

Recent studies have reported that miRNAs are involved in the process of inflammatory response. miR-126 is an endothelial cell-specific miRNA. It targets to VCAM-1, which regulates leukocyte trafficking to sites of inflammation, and suppresses the VCAM-1 expression. Decreased miR-126 leads to an increase in the TNF-stimulated VCAM-1 expression and promotes leukocyte adherence to endothelial cells consequently [45]. miR-21 is also involved in the modulation of vascular inflammatory response. miR-21 is abundantly expressed in inflammatory cells. Loss of miR-21 represses macrophage activation, therefore reduces inflammatory response after vascular injury aroused by stent implantation [46]. Both miR-221/222 and miR-155 can also regulate endothelial inflammation by regulating endothelial adhesion molecules. Overexpression of miR-155 and miR-221/222 downregulated ETS-1, a critical transcription factor of vascular inflammation and remodeling, and its downstream signaling VCAM1, MCP-1, and FLT1, thus attenuate the adhesion of Jurkat T cells to ECs [47]. Additionally, miR-195 reduced synthesis of proinflammatory biomarkers, IL-1*β*, IL-6, and IL-8, suggesting that it could be a potential regulator of ISR because of its anti-inflammatory effect [22].

## 6. miRNAs Involved in Endothelial Regeneration

Endothelium, the inner layer of the integrated vessel wall, acts as a selectively permeable barrier between the rest of the vessel wall and blood flow and participates in regulation of vascular tone and suppression of neointimal hyperplasia by inhibiting inflammation, thrombus formation, and VSMC proliferation and migration. Compared to angioplasty, stent implantation results in severe injury because the metal scaffold provides a nonphysiological surface for adhesion and generates perturbations in blood flow and hinders the following reendothelialization as well. The reendothelialization, that is the regeneration and regrowth of the denuded endothelium, retrieved mainly from the remaining endothelial cells. However, this process after stent implantation is found to be inadequate in both structural and functional integrity with impaired endothelium-dependent vasodilation and increased permeability [11, 48].

Endothelial denudation acts as a trigger of ISR; the consequent delayed endothelial recovery and endothelial dysfunction are the major contributing factors of late stent thrombosis [49]. The antiproliferative drugs of DES have nonselective effect on both SMC and EC, thus prevent both neointimal hyperplasia and reendothelialization. Taking together, these observations confirmed the essential role of endothelium in the process of ISR, implied that promoting rather than blocking the healing process by stimulating reendothelialization seems the most natural approach to prevent restenosis after DES implantation. Experimental studies showed that delivery of miR-140-3p into rat carotid artery after balloon angioplasty leads to the reduction of neointimal hyperplasia; meanwhile, it has no adverse effect on reendothelialization. Similarly, anti-miR-21 effectively attenuates neointimal hyperplasia and ISR without impeding reendothelialization, and anti-miR-21 did not inhibit EC proliferation in vitro [50]. Thus, application of miRNAs might be a novel, cell-specific perspective in this challenging problem.

miR-126 is an EC-specific microRNA. It has been proved that miR-126 plays an irreplaceable role in the maintenance of endothelial integrity and angiogenesis. miR-126 mutant mice express severe systemic edema, multifocal hemorrhages, ruptured blood vessel, and partial embryonic lethality [51]. miR-126 targets to Spred-1, a negative regulator of MAPK signaling, therefore promotes VEGF- and FGF-mediated endothelial cell migration and angiogenesis by repressing Spred-1. Although miR-126 is not expressed in VSMCs, it participates in the regulation of VSMC function. By targeting and downregulating insulin receptor substrate-1 (IRS-1), miR-126 reduces VSMC proliferation and migration. Moreover, Taglin and Acta2, which indicate VSMC differentiation, were upregulated by miR-126 in VSMCs [52].

miR-92a is another essential mediator of endothelial functions and angiogenesis which is selectively expressed in EC but not in SMC [53]. Inhibition of miR-92a induced increased phosphorylation of ERK1/2 and c-jun N-terminal kinases/stress-activated protein kinases (JNK/SAPK), as well as increased serum response factor (SRF) protein level, and therefore enhances EC proliferation and migration in vitro. Moreover, inhibition of miR-92a also induced increased Klf-4 expression, which regulates endothelial hemostasis, and thus enhances the eNOS expression and its nitric oxide (NO) production in EC. Not only maintain the endothelial integrity, the EC released NO also negatively regulates SMC proliferation and migration and consequently inhibits neointimal hyperplasia [54]. Importantly, functional inhibition of miR-92a in vivo prominently reduced neointimal hyperplasia and accelerated the reendothelialization after vascular injury and stent implantation, indicating that regulation of miR-92a could be a novel therapeutic approach to prevent ISR [53]. miR-92 is a member of the miR17-92 cluster, a polycistronic unit encoding miR-17, miR-18a, miR-19a/b, miR-20a, and miR-92a. All the members of the miR17-92 cluster are expressed in EC and known to participate in VSMC proliferation and neointimal hyperplasia of carotid artery restenosis [55]. miR17-92 cluster members are upregulated in carotid artery restenosis; they mediate the crosstalk of two TGF-*β* signaling pathway, smad3 and bone morphogenetic protein receptor type II (BMPR2). Smad3 activates miR17-92, therefore downregulated BMPR2 which directly target to miR17-92, and consequently promotes neointimal hyperplasia and carotid artery restenosis [55].

miR221 and miR222 are highly expressed in both EC and VSMC. In VSMC, miR221 and miR222 exhibit strong proproliferative effects. After vascular injury induced by angioplasty, the miR221 and miR222 expressions are increased and promote VSMC proliferation and neointimal hyperplasia via inhibits their target gene p27 (Kip1) and p57 (Kip2) [56]. On the contrary, in EC, upregulated miR221 and miR222 suppress EC proliferation/migration, promote EC apoptosis, and lead to decreased reendothelialization after angioplasty via inhibiting c-kit, which expressed in EC, but not VSMC [57]. The opposite effects of miR221 and miR222 expression on EC and VSMC may partly due to differential target gene expression in different cell types. And this imply that inhibit miR221 and miR222 could repress neointimal hypoplasia while enhance reendothelialization, which are responsible for ISR reducing.

miR-143/-145 is undetectable in endothelial cells; however, its expression increased in response to shear stress and Klf-2, the shear-responsive transcription factor. It has been proved that Klf-2 expressed endothelial cell transfer miR-143/-145 enriched extracellular vesicles to SMCs, influencing SMC functions in a paracrine manner [58]. Furthermore, when coculture VSMC and EC, the cell-to-cell contact induces miR-143/-145 transfer from SMC to EC via tunneling nanotubes [59]. These cell-to-cell crosstalk studies illustrate that miR-143/-145 acts as a signaling transmitter to communicate VSMCs and ECs and regulate their functions consequently.

## 7. Interaction with lncRNA in the Vasculature

Long noncoding RNAs (lncRNAs) are nonprotein coding transcripts with a length of more than 200 nt. lncRNAs are highly versatile; they can be regulated by miRNA and the reciprocal. Generally, the mechanism of microRNAs and lncRNA interactions can be summarized in 3 ways [60]. First, lncRNAs bind and compete with microRNAs (lncRNA-microRNA sponge) to reduce microRNA function with cells and alleviate mRNA repressions. Besides, lncRNAs host microRNAs within their exons and introns. Finally, microRNAs bind lncRNAs and regulate their stability.

LncRNA 362 was identified as one of the angiotensin II- (Ang II-) responsive RNAs [61], and it acts as host gene for miR-221 and miR-222 which has been identified as regulators of VSMC proliferation and migration [56]. Knocking-down of LncRNA 362 results in decreased miR-221/222 expression and subsequent inhibition of VSMC proliferation. It suggested that the coregulation of LncRNA362 and miR-221/222 could promote VSMC proliferation and neointimal hyperplasia [60, 62].

LncRNA-H19 modulates let-7 availability, which have been indicated to protect VSMC from oxidative damage, by acting as a molecular sponge [63, 64]. Also, LncRNA-H19 is a primary precursor for miR-675. It has been proved that both of LncRNA-H19 and miR-675 expression are increased after vascular injury. LncRNA-H19 accelerates VSMC proliferation in a miR-675 dependent manner by directly targets to PTEN [65].

LncRNA LINC00341 acts as the sponge of miR-214 and therefore promotes its target protein FOXO4 expression, and transcription factor FOXO4 activates the transcription of LINC00341. LINC00341 promotes the proliferation and migration of VSMCs via this positive feedback loop [66]. Tian et al.'s study revealed that the lncRNA UCA1, which is known to play an important role in cardiovascular injury, acts as a sponge of miR-26a and downregulates miR-26a expression, thereby alleviates smooth muscle cell proliferation against atherosclerosis [67]. Recently, Zhang et al. discovered that the lncRNA POU3F3 expression increased in ISR patients after PCI. POU3F3 promotes VSMC proliferation, migration, and phenotypic transformation via POU3F3/miR-449a/KLF4 signaling pathway [68].

LncRNA MALAT1 expressed in ECs, and it reciprocally interacts with miR-320a. In human umbilical vein endothelial cells (HUVECs), knockdown of MALAT1 induced increasing of miR-320a expression and decreasing of FOXM1 expression, consequently inhibited HUVEC proliferation [69].

## 8. Circulating miRNAs as Biomarkers for ISR

miRNAs can be found in the blood circulation in an extraordinary stable form, and they are susceptible to sensitive detection through qPCR, thus were identified as potential biomarkers of many cardiovascular disease, predicting the response of therapeutic interventions, such as ISR [70].

A case-control study reveals that circulating miR-21 level increased while miR-100, miR143, and miR145 decreased in patients with ISR compared to healthy people and non-ISR patient and demonstrate these miRNAs as potential biomarkers of ISR. Interestingly, they can discriminate between diffuse ISR and local ISR [71]. miR-93-5p was able to discriminate between patients with stable coronary artery disease (CAD) and those with no CAD; furthermore, it is a powerful independent predictor of coronary ISR [72]. A recent study found that miR-19a, miR-126, miR-210, and miR-378 independently correlated with lower restenosis occurrence, demonstrating that these four miRNAs had good value in predicting restenosis risk [73].

There are also several studies performed to explore the predict value of miRNAs in PAD restenosis. Circulating microRNA-320a and microRNA-572 in PAD patients with ISR had significantly higher expression levels than it from non-ISR and healthy volunteers [74]. miR-195 is a PAD-specific miRNA that has potential diagnostic value to discriminate patients with PAD from healthy population [75]. Moreover, it is a strong and independent predictor of adverse atherothrombotic events and target vessel revascularization following angioplasty and stenting for PAD, and it could become a valuable and easily accessible biomarker for risk stratification after endovascular revascularization procedures [76].

## 9. Future Perspectives and Conclusion

The invasive endovascular repair including PTA and stent implantation has become the standard approach for both CAD and PAD. Despite the development of DES significantly reduced the rate of restenosis compared to BMS and PTA alone, ISR is still the major drawback of endovascular repair. DES failed to completely obliterate ISR, and it induced risk of late stent thrombosis, hindered endothelial healing and inflammatory response due to nonselective inhibition of endothelial and SMC proliferation by the antiproliferative drugs [77]. Therefore, based on the comprehensive understanding of ISR pathophysiology, an ideal therapeutic target to prevent ISR should able to inhibit SMC proliferation induced neointimal hyperplasia and facilitate endothelial healing simultaneously.

The recent development of gene-eluting stent is a major breakthrough in stent technology. Paul and colleagues developed a nanobiohybrid hydrogel-based endovascular stent device to prevent ISR [78]. In this study, the hydrogel works as a reservoir to carry, protect, and simultaneously deliver proangiogenic, VEGF, and Angiopoietin-1 genes to the target site. And the results showed enhancement in reendothelialization, attenuation of stenosis, and prevention of neointimal formation. Yang et al. reported a stent coated with bilayered poly (lactide-co-glycolide) (PLGA) nanoparticles containing VEGF plasmid in the outer layer and paclitaxel in the inner core (VEGF/PTX NPs), demonstrating that the VEGF/PTX NP-coated stent promotes early endothelium healing while inhibits SMC proliferation through sequential release of the VEGF gene and paclitaxel, resulted in significant suppression of ISR [79]. These studies inspired us that restenosis could be prevented at the molecular level by administering gene expression, and miRNA-eluting stent could be a promising therapy because miRNA can inhibit ISR via more than one pathway. One of the challenging puzzles is choosing appropriate target miRNA. The miR-195, miR-92a, and miR-221/222 are potential targets of the miRNA-eluting stent, because miR-195 effectively prevents ISR by inhibiting VSMC proliferation and migration while reducing inflammatory response as well, whereas miR-92a and miR-221/222 showed different effects on VSMCs and ECs.

As for miRNA-based therapy, silencing miR-122 leads to long-lasting suppression of hepatitis C virus (HCV) viremia maybe be the first human therapy [80]. However, compared to reach up to liver, specific miRNA delivery to the vascular system can be more challenging. Santulli et al. used an adenoviral vector that encodes p27 with target sequences for EC-specific miR-126-3p at the 3′ end, demonstrating the potential of using a miRNA-based strategy as a therapeutic approach to specifically inhibit vascular restenosis while preserving EC function [81]. Wang et al. reported application of anti-21-coated stents effectively reduced ISR, whereas no significant off-target effects could be observed [50].

Despite the therapeutic implication of some miRNA in restenosis has entered clinical trials by integrating them in microsphere-integrated stent, adenovirus, or nanoparticles [82], there are still many problems need to be solved. For example, since miRNA controls multiple pathways in different tissue types, how to avoid the unwanted effects accompanied with miRNA inhibition is of importance. Some miRNAs might have an ambivalent effect that is both a beneficial therapeutic effect in terms of angiogenesis and a detrimental promotion of tumor angiogenesis.

In conclusion, miRNAs exhibit great therapeutic potential for ISR treatment. Fully understand the molecular mechanisms underlying miRNA regulated ISR is of paramount importance to promote the realization of miRNA-based strategy, despite there is still a long way to go before clinical application of miRNA-eluting stent.

## Figures and Tables

**(a):** 

miRNAs	Expressed cell type	Up (↑) and down (↓) regulation after vascular injury	Function regulated	Target gene(s)	Preclinical model	Ref
miR-143/145	VSMC	↓	Differentiation, migration, proliferation	Klf-4, Klf-5, Elk-1	Carotid artery balloon injury model in Sprague-Dawley rat	[19, 21]
miR-195	VSMC	↓	Proliferation, migration	Cdc-42, CCND1	Carotid artery balloon injury model in Sprague-Dawley rat	[22]
miR-125	VSMC	↓	Proliferation, migration	ETS-1, MAPK1	Carotid artery balloon injury model in Sprague-Dawley rat and Wistar rat	[23, 25]
miR-23b	VSMC	↓	Proliferation, migration	uPA, Smad3, FOXO4	Carotid artery balloon injury model in Wistar rat	[27]
miR-663	VSMC	↓	Proliferation, migration	JunB, Myl9	Carotid artery ligation injury model in C57BL/6N mice	[30]
miR-140-3p	VSMC	↓	Proliferation, apoptosis	c-Myb, Bcl-2	Carotid artery restenosis model in Sprague-Dawley rat	[31]
miR-22-3p	VSMC	↓ (in ASO)	Proliferation, migration	HMGB1	Carotid artery balloon injury model in rat	[32]
miR-1298	VSMC	↓ (in ASO)	Proliferation, migration	Cx43	Carotid artery balloon injury model in rat	[33]
miR-21	VSMC	↑	Proliferation, migration, apoptosis	PTEN, Bcl-2	Thoracic aorta stent implantation in mice	[35, 46]

**(b):** 

miRNAs	Expressed cell type	Up (↑) and down (↓) regulation after vascular injury	Function regulated	Target gene(s)	Preclinical model	Ref
miR-146	VSMC	↑	Proliferation	Klf-4, Klf-5	Carotid artery balloon injury model in rat	[37]
miR-424/322	VSMC	↑	Proliferation, migration, apoptosis	CCND1, STIM1	Carotid artery balloon injury model in rat	[38]
miR-133	VSMC	↓ (in early phase), ↑ (in late phase)	Proliferation, migration	Sp-1, moesin	Carotid artery balloon injury model in rat	[39]
miR-132	VSMC	↓ (in early phase), ↑ (in late phase)	Proliferation, migration, apoptosis, survival	LRRFIP1	Carotid artery injury model in rat	[40]
miR-638	VSMC	—	Proliferation, migration	NOR1	—	[41]
miR-126	EC	—	Proliferation, migration	Spred-1, VCAM-1	Iliac artery neointimal formation model in rabbit	[51]
miR-92a	EC	↑	Proliferation, migration, NO release	BMPR2, Klf-4, MKK4	Carotid artery balloon injury model in Wistar rat	[53, 55]
miR-221/222	VSMC and EC	↑	Proliferation in VSMC, proliferation, migration, apoptosis in EC	p27 (Kip1), p57 (Kip2), c-kit, ETS-1, ICAM-1	Carotid artery balloon injury model in rat	[47, 56, 57]

## Data Availability

All relevant data are within the paper and its supporting information files.

## Abbreviations

3-UTRs: 3′-Untranslated regions
ACE: Angiotensin converting enzyme
ACTA2: Alpha Smooth Muscle Actin 2
ASO: Arteriosclerosis obliterans
BMPR2: Bone morphogenetic protein receptor type II
BMS: Bare metal stent
CAD: Coronary artery disease
CCND1: CyclinD1
DES: Drug-eluted stent
ECs: Endothelial cells
ECM: Extracellular matrix
ETS-1: E26 transformation specific-1
FGF: Fibroblast growth factor
FOXO4: Forkhead box O4
HCV: Hepatitis C virus
HMGB-1: High mobility group box-1
ICAM-1: Intercellular adhesion molecule-1
IL: Interleukin
IRS-1: Insulin receptor substrate-1
ISR: In-stent restenosis
JNK/SAPK: c-Jun N-terminal kinases/stress-activated protein kinases
Klf: Kruppel-like factor
lncRNA: Long noncoding RNA
LRRFIP1: Leucine-rich repeat (in Flightless 1) interacting protein-1
MCP-1: Monocyte chemoattractant protein-1
MAPK: Mitogen-activated Protein Kinase
miRNA: MicroRNAs
miRISC: miRNA-induced silencing complex
MREs: miRNA-response elements
MRTF-*β*: Myocardin-related transcription factor-beta
MYH11: Myosin Heavy Chain 11
NO: Nitric oxide
PAD: Peripheral arterial disease
PDGF: Platelet-derived growth factor
PLGA: Poly(lactide-co-glycolide)
PTA: Percutaneous transluminal angioplasty
PTEN: Phosphatase and tensin homolog
SM22*α*: Smooth Muscle 22 alpha
SM-MHC: Smooth muscle myosin heavy chain
SRF: Serum response factor
STIM1: Stromal interaction molecule 1
TGF-*β*: Transforming growth factor-beta
uPA: Urokinase plasminogen activator
VCAM-1: Vascular adhesion molecules-1
VEGF: Vascular endothelial growth factor
VSMCs: Vascular smooth muscle cells.

## References

[1] Norgren L., Hiatt W. R., Dormandy J. A., Nehler M. R., Harris K. A., Fowkes F. G. R. (2007). Inter-Society Consensus for the Management of Peripheral Arterial Disease (TASC II). *J Vasc Surg*.

[2] Shammas N. W. (2007). Epidemiology, classification, and modifiable risk factors of peripheral arterial disease. *Vascular Health and Risk Management*.

[3] Ho K. J., Owens C. D. (2017). Diagnosis, classification, and treatment of femoropopliteal artery in-stent resten1o4../8sis. *Journal of Vascular Surgery*.

[4] Abizaid A., Kornowski R., Mintz G. S. (1998). The influence of diabetes mellitus on acute and late clinical outcomes following coronary stent implantation. *Journal of the American College of Cardiology*.

[5] Papafaklis M. I., Chatzizisis Y. S., Naka K. K., Giannoglou G. D., Michalis L. K. (2012). Drug-eluting stent restenosis: effect of drug type, release kinetics, hemodynamics and coating strategy. *Pharmacology & Therapeutics*.

[6] Costa M. A., Simon D. I. (2005). Molecular basis of restenosis and drug-eluting stents. *Circulation*.

[7] Welt F. G., Rogers C. (2002). Inflammation and restenosis in the stent era. *Arteriosclerosis, Thrombosis, and Vascular Biology*.

[8] Inoue T., Sakai Y., Hoshi K., Yaguchi I., Fujito T., Morooka S. (1998). Lower expression of neutrophil adhesion molecule indicates less vessel wall injury and might explain lower restenosis rate after cutting balloon angioplasty. *Circulation*.

[9] Mitra A. K., Agrawal D. K. (2006). In stent restenosis: bane of the stent era. *Journal of Clinical Pathology*.

[10] Riessen R., Isner J. M., Blessing E., Loushin C., Nikol S., Wight T. N. (1994). Regional differences in the distribution of the proteoglycans biglycan and decorin in the extracellular matrix of atherosclerotic and restenotic human coronary arteries. *The American Journal of Pathology*.

[11] Cornelissen A., Vogt F. J. (2019). The effects of stenting on coronary endothelium from a molecular biological view: time for improvement?. *Journal of Cellular and Molecular Medicine*.

[12] Wronska A., Kurkowska-Jastrzebska I., Santulli G. (2015). Application of microRNAs in diagnosis and treatment of cardiovascular disease. *Acta Physiologica (Oxford, England)*.

[13] De Rosa S., Curcio A., Indolfi C. (2014). Emerging role of microRNAs in cardiovascular diseases. *Circulation Journal*.

[14] Stellos K., Dimmeler S. (2014). Vascular microRNAs: from disease mechanisms to therapeutic targets. *Circulation Research*.

[15] Gareri C., De Rosa S., Indolfi C. (2016). MicroRNAs for restenosis and thrombosis after vascular injury. *Circulation Research*.

[16] Kearney M., Pieczek A., Haley L. (1997). Histopathology of in-stent restenosis in patients with peripheral artery disease. *Circulation*.

[17] Owens G. K. (1995). Regulation of differentiation of vascular smooth muscle cells. *Physiological Reviews*.

[18] Owens G. K., Kumar M. S., Wamhoff B. R. (2004). Molecular regulation of vascular smooth muscle cell differentiation in development and disease. *Physiological Reviews*.

[19] Cordes K. R., Sheehy N. T., White M. P. (2009). miR-145 and miR-143 regulate smooth muscle cell fate and plasticity. *Nature*.

[20] Zhao W., Zhao S. P., Zhao Y. H. (2015). MicroRNA-143/-145 in cardiovascular diseases. *BioMed Research International*.

[21] Cheng Y., Liu X., Yang J. (2009). MicroRNA-145, a novel smooth muscle cell phenotypic marker and modulator, controls vascular neointimal lesion formation. *Circulation Research*.

[22] Wang Y. S., Wang H. Y., Liao Y. C. (2012). MicroRNA-195 regulates vascular smooth muscle cell phenotype and prevents neointimal formation. *Cardiovascular Research*.

[23] Gareri C., Iaconetti C., Sorrentino S., Covello C., De Rosa S., Indolfi C. (2017). miR-125a-5p modulates phenotypic switch of vascular smooth muscle cells by targeting ETS-1. *Journal of Molecular Biology*.

[24] Dandré F., Owens G. K. (2004). Platelet-derived growth factor-BB and Ets-1 transcription factor negatively regulate transcription of multiple smooth muscle cell differentiation marker genes. *American Journal of Physiology. Heart and Circulatory Physiology*.

[25] Hu W., Chang G., Zhang M. (2019). MicroRNA-125a-3p affects smooth muscle cell function in vascular stenosis. *Journal of Molecular and Cellular Cardiology*.

[26] Bang C., Fiedler J., Thum T. (2012). Cardiovascular importance of the microRNA-23/27/24 family. *Microcirculation*.

[27] Iaconetti C., De Rosa S., Polimeni A. (2015). Down-regulation of miR-23b induces phenotypic switching of vascular smooth muscle cells in vitro and in vivo. *Cardiovascular Research*.

[28] Kiyan Y., Limbourg A., Kiyan R. (2012). Urokinase receptor associates with myocardin to control vascular smooth muscle cells phenotype in vascular disease. *Arteriosclerosis, Thrombosis, and Vascular Biology*.

[29] Tsai S., Hollenbeck S. T., Ryer E. J. (2009). TGF-*β* through Smad3 signaling stimulates vascular smooth muscle cell proliferation and neointimal formation. *American Journal of Physiology-Heart and Circulatory Physiology*.

[30] Li P., Zhu N., Yi B. (2013). MicroRNA-663 regulates human vascular smooth muscle cell phenotypic switch and vascular neointimal formation. *Circ Res*.

[31] Zhu Z. R., He Q., Wu W. B. (2018). MiR-140-3p is involved in in-stent restenosis by targeting C-Myb and BCL-2 in peripheral artery disease. *Journal of Atherosclerosis and Thrombosis*.

[32] Huang S. C., Wang M., Wu W. B. (2017). Mir-22-3p inhibits arterial smooth muscle cell proliferation and migration and neointimal hyperplasia by targeting HMGB1 in arteriosclerosis obliterans. *Cellular Physiology and Biochemistry*.

[33] Hu W., Wang M., Yin H. (2015). MicroRNA-1298 is regulated by DNA methylation and affects vascular smooth muscle cell function by targeting connexin 43. *Cardiovascular Research*.

[34] Chadjichristos C. E., Morel S., Derouette J. P. (2008). Targeting connexin 43 prevents platelet-derived growth factor-BB-induced phenotypic change in porcine coronary artery smooth muscle cells. *Circulation Research*.

[35] Ji R., Cheng Y., Yue J. (2007). MicroRNA expression signature and antisense-mediated depletion reveal an essential role of MicroRNA in vascular neointimal lesion formation. *Circulation Research*.

[36] Wang M., Li W., Chang G. Q. (2011). MicroRNA-21 regulates vascular smooth muscle cell function via targeting tropomyosin 1 in arteriosclerosis obliterans of lower extremities. *Arteriosclerosis, Thrombosis, and Vascular Biology*.

[37] Sun S. G., Zheng B., Han M. (2011). miR-146a and Krüppel-like factor 4 form a feedback loop to participate in vascular smooth muscle cell proliferation. *EMBO Reports*.

[38] Merlet E., Atassi F., Motiani R. K. (2013). miR-424/322 regulates vascular smooth muscle cell phenotype and neointimal formation in the rat. *Cardiovascular Research*.

[39] Torella D., Iaconetti C., Catalucci D. (2011). MicroRNA-133 controls vascular smooth muscle cell phenotypic switch in vitro and vascular remodeling in vivo. *Circulation Research*.

[40] Choe N., Kwon J. S., Kim J. R. (2013). The microRNA miR-132 targets Lrrfip1 to block vascular smooth muscle cell proliferation and neointimal hyperplasia. *Atherosclerosis*.

[41] Li P., Liu Y., Yi B. (2013). MicroRNA-638 is highly expressed in human vascular smooth muscle cells and inhibits PDGF-BB-induced cell proliferation and migration through targeting orphan nuclear receptor NOR1. *Cardiovascular Research*.

[42] Moreno P. R., Bernardi V. H., Lo´pez-Cue´llar J. (1996). Macrophage infiltration predicts restenosis after coronary intervention in patients with unstable angina. *Circulation*.

[43] Cipollone F., Marini M., Fazia M. (2001). Elevated circulating levels of monocyte chemoattractant protein-1 in patients with restenosis after coronary angioplasty. *Arteriosclerosis, Thrombosis, and Vascular Biology*.

[44] Tanaka H., Sukhova G. K., Swanson S. J. (1993). Sustained activation of vascular cells and leukocytes in the rabbit aorta after balloon injury. *Circulation*.

[45] Harris T. A., Yamakuchi M., Ferlito M., Mendell J. T., Lowenstein C. J. (2008). MicroRNA-126 regulates endothelial expression of vascular cell adhesion molecule 1. *Proceedings of the National Academy of Sciences of the United States of America*.

[46] McDonald R. A., Halliday C. A., Miller A. M. (2015). Reducing in-stent restenosis: therapeutic manipulation of miRNA in vascular remodeling and inflammation. *Journal of the American College of Cardiology*.

[47] Zhu N., Zhang D., Chen S. (2011). Endothelial enriched microRNAs regulate angiotensin II-induced endothelial inflammation and migration. *Atherosclerosis*.

[48] van Beusekom H. M., Whelan D. M., Hofma S. H. (1998). Long-term endothelial dysfunction is more pronounced after stenting than after balloon angioplasty in porcine coronary arteries. *Journal of the American College of Cardiology*.

[49] Joner M., Finn A. V., Farb A. (2006). Pathology of drug-eluting stents in humans: delayed healing and late thrombotic risk. *Journal of the American College of Cardiology*.

[50] Wang D., Deuse T., Stubbendorff M. (2015). Local microRNA modulation using a novel anti-miR-21-eluting stent effectively prevents experimental in-stent restenosis. *Arteriosclerosis, Thrombosis, and Vascular Biology*.

[51] Wang S., Aurora A. B., Johnson B. A. (2008). The endothelial-specific microRNA miR-126 governs vascular integrity and angiogenesis. *Developmental Cell*.

[52] Izuhara M., Kuwabara Y., Saito N. (2017). Prevention of neointimal formation using miRNA-126-containing nanoparticle-conjugated stents in a rabbit model. *PLoS One*.

[53] Iaconetti C., Polimeni A., Sorrentino S. (2012). Inhibition of miR-92a increases endothelial proliferation and migration in vitro as well as reduces neointimal proliferation in vivo after vascular injury. *Basic Research in Cardiology*.

[54] Indolfi C., Torella D., Coppola C. (2002). Physical training increases eNOS vascular expression and activity and reduces restenosis after balloon angioplasty or arterial stenting in rats. *Circulation Research*.

[55] Luo T., Cui S., Bian C., Yu X. (2014). Crosstalk between TGF-*β*/Smad3 and BMP/BMPR2 signaling pathways via miR-17-92 cluster in carotid artery restenosis. *Molecular and Cellular Biochemistry*.

[56] Liu X., Cheng Y., Zhang S., Lin Y., Yang J., Zhang C. (2009). A necessary role of miR-221 and miR-222 in vascular smooth muscle cell proliferation and neointimal hyperplasia. *Circulation Research*.

[57] Liu X., Cheng Y., Yang J., Xu L., Zhang C. (2012). Cell-specific effects of miR-221/222 in vessels: molecular mechanism and therapeutic application. *Journal of Molecular and Cellular Cardiology*.

[58] Hergenreider E., Heydt S., Tréguer K. (2012). Atheroprotective communication between endothelial cells and smooth muscle cells through miRNAs. *Nature Cell Biology*.

[59] Climent M., Quintavalle M., Miragoli M., Chen J., Condorelli G., Elia L. (2015). TGF*β* triggers miR-143/145 transfer from smooth muscle cells to endothelial Cells, Thereby Modulating Vessel Stabilization. *Circulation Research*.

[60] Ballantyne M. D., McDonald R. A., Baker A. H. (2016). lncRNA/MicroRNA interactions in the vasculature. *Circulation Research*.

[61] Leung A., Trac C., Jin W. (2013). Novel long noncoding RNAs are regulated by angiotensin II in vascular smooth muscle cells. *Circulation Research*.

[62] Li H., Zhu H., Ge J., Noncoding R. N. A. (2016). Long Noncoding RNA: Recent updates in atherosclerosis. *International Journal of Biological Sciences*.

[63] Ding Z., Wang X., Schnackenberg L. (2013). Regulation of autophagy and apoptosis in response to ox-LDL in vascular smooth muscle cells, and the modulatory effects of the microRNA hsa-let-7g. *International Journal of Cardiology*.

[64] Kallen A. N., Zhou X. B., Xu J. (2013). The imprinted H19 lncRNA antagonizes let-7 microRNAs. *Molecular Cell*.

[65] Lv J., Wang L., Zhang J. (2018). Long noncoding RNA H19-derived miR-675 aggravates restenosis by targeting PTEN. *Biochemical and Biophysical Research Communications*.

[66] Liu X., Ma B. D., Liu S., Liu J., Ma B. X. (2019). Long noncoding RNA LINC00341 promotes the vascular smooth muscle cells proliferation and migration via miR-214/FOXO4 feedback loop. *American Journal of Translational Research*.

[67] Tian S., Yuan Y., Li Z., Gao M., Lu Y., Gao H. (2018). LncRNA UCA1 sponges miR-26a to regulate the migration and proliferation of vascular smooth muscle cells. *Gene*.

[68] Zhang J., Gao F., Ni T. (2019). Linc-POU3F3 is overexpressed in in-stent restenosis patients and induces VSMC phenotypic transformation via POU3F3/miR-449a/KLF4 signaling pathway. *American Journal of Translational Research*.

[69] Sun J. Y., Zhao Z. W., Li W. M. (2017). Knockdown of MALAT1 expression inhibits HUVEC proliferation by upregulation of miR-320a and downregulation of FOXM1 expression. *Oncotarget*.

[70] Varela N., Lanas F., Salazar L. A., Zambrano T. (2020). The current state of microRNAs as restenosis biomarkers. *Frontiers in Genetics*.

[71] He M., Gong Y., Shi J. (2014). Plasma microRNAs as potential noninvasive biomarkers for in-stent restenosis. *PLoS One*.

[72] O'Sullivan J. F., Neylon A., Fahy E. F., Yang P., McGorrian C., Blake G. J. (2019). MiR-93-5p is a novel predictor of coronary in-stent restenosis. *Heart Asia*.

[73] Dai R., Liu Y., Zhou Y. (2020). Potential of circulating pro-angiogenic microRNA expressions as biomarkers for rapid angiographic stenotic progression and restenosis risks in coronary artery disease patients underwent percutaneous coronary intervention. *Journal of Clinical Laboratory Analysis*.

[74] Yuan L., Dong J., Zhu G. (2019). Diagnostic value of circulating microRNAs for in-stent restenosis in patients with lower extremity arterial occlusive disease. *Scientific Reports*.

[75] Stather P. W., Sylvius N., Wild J. B., Choke E., Sayers R. D., Bown M. J. (2013). Differential microRNA expression profiles in peripheral arterial disease. *Circulation: Cardiovascular Genetics*.

[76] Stojkovic S., Jurisic M., Kopp C. W. (2018). Circulating microRNAs identify patients at increased risk of in-stent restenosis after peripheral angioplasty with stent implantation. *Atherosclerosis*.

[77] Iakovou I., Schmidt T., Bonizzoni E. (2005). Incidence, predictors, and outcome of thrombosis after successful implantation of drug-eluting stents. *Journal of the American Medical Association*.

[78] Paul A., Shao W., Shum-Tim D., Prakash S. (2012). The attenuation of restenosis following arterial gene transfer using carbon nanotube coated stent incorporating TAT/DNA_Ang1+Vegf_ nanoparticles. *Biomaterials*.

[79] Yang J., Zeng Y., Zhang C. (2013). The prevention of restenosis in vivo with a VEGF gene and paclitaxel co- eluting stent. *Biomaterials*.

[80] Lanford R. E., Hildebrandt-Eriksen E. S., Petri A. (2010). Therapeutic silencing of microRNA-122 in primates with chronic hepatitis C virus infection. *Science*.

[81] Santulli G., Wronska A., Uryu K. (2014). A selective microRNA-based strategy inhibits restenosis while preserving endothelial function. *Journal of Clinical Investigation*.

[82] Liu S., Yang Y., Jiang S. (2018). Understanding the role of non-coding RNA (ncRNA) in stent restenosis. *Atherosclerosis*.

